# Sevoflurane Alters Spatiotemporal Functional Connectivity Motifs That Link Resting-State Networks during Wakefulness

**DOI:** 10.3389/fncir.2016.00107

**Published:** 2016-12-27

**Authors:** MohammadMehdi Kafashan, ShiNung Ching, Ben J. A. Palanca

**Affiliations:** ^1^Department of Electrical and Systems Engineering, Washington University in St. LouisSt. Louis, MO, USA; ^2^Division of Biology and Biomedical Science, Washington University in St. LouisSt. Louis, MO, USA; ^3^Department of Anesthesiology, Washington University School of Medicine in St. LouisSt. Louis, MO, USA

**Keywords:** resting-state functional MRI, dynamic functional connectivity, sevoflurane, spatiotemporal analysis, Kalman filtering

## Abstract

**Background:** The spatiotemporal patterns of correlated neural activity during the transition from wakefulness to general anesthesia have not been fully characterized. Correlation analysis of blood-oxygen-level dependent (BOLD) functional magnetic resonance imaging (fMRI) allows segmentation of the brain into resting-state networks (RSNs), with functional connectivity referring to the covarying activity that suggests shared functional specialization. We quantified the persistence of these correlations following the induction of general anesthesia in healthy volunteers and assessed for a dynamic nature over time.

**Methods:** We analyzed human fMRI data acquired at 0 and 1.2% vol sevoflurane. The covariance in the correlated activity among different brain regions was calculated over time using bounded Kalman filtering. These time series were then clustered into eight orthogonal motifs using a K-means algorithm, where the structure of correlated activity throughout the brain at any time is the weighted sum of all motifs.

**Results:** Across time scales and under anesthesia, the reorganization of interactions between RSNs is related to the strength of dynamic connections between member pairs. The covariance of correlated activity between RSNs persists compared to that linking individual member pairs of different RSNs.

**Conclusions:** Accounting for the spatiotemporal structure of correlated BOLD signals, anesthetic-induced loss of consciousness is mainly associated with the disruption of motifs with intermediate strength within and between members of different RSNs. In contrast, motifs with higher strength of connections, predominantly with regions-pairs from within-RSN interactions, are conserved among states of wakefulness and sevoflurane general anesthesia.

## 1. Introduction

The induction of general anesthesia incurs a dramatic change in phenotype compared to wakefulness. Substantial progress has been made at identifying the molecular targets (Franks, 2008) and subcortical arousal systems (Schwartz et al., 2010) perturbed in this transition. Correlated electroencephalographic activity among distributed scalp regions also changes substantively, suggesting concurrent perturbation in the networks underlying cognition and behavior (Jordan et al., 2013; Akeju et al., 2014a,b; Blain-Moraes et al., 2014, 2015; Kaskinoro et al., 2015; Purdon et al., 2015). While prior investigations have evaluated the temporal dynamics of electroencephalographic signatures during anesthetized states (Chander et al., 2014; Hudson et al., 2014), the characterization of fluctuations in neural activity across both space and time have been lacking. Functional magnetic resonance imaging (fMRI) studies have shown that the temporal variability in the blood-oxygen-level dependent (BOLD) signal varies between wakefulness and general anesthesia (Huang et al., 2014, 2016).

The disruption of resting-state networks (RSNs) is a candidate mechanism whereby anesthetic agents induce sedation and unconsciousness. Each RSN encompasses brain regions of presumably shared functional specialization that demonstrate marked zero time lag correlation among surrogates of neural activity. This phenomenon of functional connectivity recapitulates the patterns of brain activation associated with particular stimulus or task. These findings evoke a complementary line of inquiring regarding which components of the functional neuroarchitecture underlying wakefulness remain preserved during the anesthetized state. General anesthesia appear to perturb RSNs in both animals (Vincent et al., 2007) and humans (Peltier et al., 2005; Boveroux et al., 2010; Palanca et al., 2015).

However, these previous approaches rely on the classical functional connectivity (FC) method of obtaining a single correlation metric over an entire recording. Such an approach obscures changes that might occur in the temporal dynamics of the FC, i.e., dynamic functional connectivity. We hypothesized that analyzing these temporal dynamics (the dynamical functional connectivity) would reveal additional anesthesia-induced disruptions within and between RSNs. While different approaches (Kang et al., 2011) have been developed, interpretability and statistical evaluation of such approaches remain unresolved (Hutchison et al., 2013a; Calhoun et al., 2014; Keilholz, 2014), and no standard consensus exists. Prior work to assess these dynamics in the anesthetized state has employed a framework wherein brain activation transitions within a discrete set of exclusive states (Barttfeld et al., 2015). However, with this approach, it is difficult to disassociate particular region pairs in terms of their temporal dynamics. In other words, certain region pairs may evolve slowly, while other evolve quickly, giving the appearance of a discrete set of states (Hutchison et al., 2013b; Amico et al., 2014; Liang et al., 2015; Thompson et al., 2015). Moreover, there are no empirical bounds on the number/types of states that exist during wakefulness and altered states of consciousness.

Extending on prior work (Kafashan et al., 2014), we studied how dynamic functional connectivity changes in subjects who underwent general anesthesia induced by the halogenated ether, sevoflurane. We sought to understand how FC among different pairs of cortical regions covary across time and arousal state. To this end, we used an unsupervised clustering method to separate the FC time series into groups of region pairs that exhibit similar dynamic functional connectivity temporal profiles—spatiotemporal motifs that exhibit similar dynamics of correlated activity that are coexpressed at each point in time. Overall, our results quantify the extent of conservation within the functional architecture of the brain despite robust pharmacological perturbation, and the identification of dynamic components that are more transient and less robust to anesthesia.

## 2. Materials and methods

### 2.1. Participants and data acquisition

Secondary analysis of data was carried out as previously described (Palanca et al., 2015). Briefly, healthy human volunteers underwent structural and functional MRI during quiet wakefulness and while rendered unresponsive by 1.2 vol% sevoflurane. Volunteers remained spontaneously ventilating by mask without assisted ventilation. Maintenance of general anesthesia and unconsciousness was assessed by lack of response to noxious finger bed pressure. Arterial carbon dioxide levels partial pressures were monitored. At least 15 min of equilibration were alloted at 1.2% sevoflurane prior to imaging. Data were acquired using a 3 Tesla Siemens Trio scanner (Siemens, Erlangen, Germany) and a 12-channel head coil. During both experimental conditions, at least two 7.5 min T2^*^-weighted scans (TR 2200 ms, TE 27 ms, FA 90 degrees, FOV 256 mm, 36 slices/volume, 200 volumes/run, 4 mm isotropic) of resting-state echoplanar BOLD images were acquired. Structural images were also acquired, including a T1-weighed (Magnetization Prepared Rapid Gradient Echo, MPRAGE: TR 2400 ms, TE 3.16 ms, FA 8 degrees, FOV 256 mm, 176 slices, 1 mm isotropic) and T2-weighted scans (TR 6280 ms, TE 88 ms, FA 120 degrees, FOV 256 mm, 36 slices, 1 × 1 × 4 mm).

### 2.2. BOLD fMRI data pre-processing

Following established pre-processing of BOLD images (Shulman et al., 2010), additional maneuvers were undertaken for magnetic field distortion correction and generation of physiologic nuisance regressors. Strict frame censoring of motion artifact (DVARS < 0.4 root-mean square BOLD signal change across adjacent image frames (Power et al., 2014) and regression of whole brain global signal were both utilized to minimize artifacts related to micromovements of the head. While global signal regression remains a controversial technique, it has been shown to be effective at reducing the spurious changes in correlated BOLD signals that are introduced by motion of the head (Power et al., 2012, 2014; Yan et al., 2013).

### 2.3. Cortical segmentation into resting-state networks

Cortical gray matter was segmented into 6 × 6 × 6 mm regions. Instead of 9 × 9 × 9 mm regions as in the prespecified analysis (Palanca et al., 2015), we used a higher spatial sampling for two reasons: (1) to maximize the likelihood of an individual voxel to include gray matter of only one RSN and (2) to maximize the spatial independence between sampled voxels. A winner-take-all algorithm selected 1076 regions with at least 50% gray matter and more than 90% probability of exclusive assignment to one of the seven RSNs. While there is no consensus for the precise brain regions associated with each RSN, we used a previously published parcellation method (Hacker et al., 2013). In this method, a supervised classifier (multi-layer perceptron) was trained to associate BOLD correlation maps corresponding to predefined seeds with specific RSN identities. This included regions of the Default Mode Network (DMN, Sestieri et al., 2010, 2011), a collection of brain areas that are more active at rest than during performing a particular task. The Dorsal Attention Network (DAN, Shulman et al., 2009; Tosoni et al., 2013), is thought to be responsible for maintaining attention to locations or features, and distinct from the network involved in detecting novel stimuli and initiating shifts of attention, referred to as the Ventral Attention Network (VAN, Corbetta et al., 2000; Astafiev et al., 2004; Kincade et al., 2005). Regions of the Frontal Parietal Control Network (FPC, Dosenbach et al., 2007) are thought to be involved in working memory and in configuring the brain's moment-to-moment information processing. Visual (VIS, Sylvester et al., 2007, 2008, 2009) and language (LAN, Sestieri et al., 2010, 2011) networks are responsible for visual and language processing, respectively. Finally, regions of the Somatomotor Network (SMN, Corbetta et al., 2000; Kincade et al., 2005; Petacchi et al., 2005) are hypothesized to be involved in active processing of motor and tactile sensory tasks. Repeated pseudorandom resampling of these regions was performed to select 15 representatives of the seven RSN for each iteration. These subsampled data of 105 voxels were used for our spatiotemporal decomposition.

### 2.4. Spatiotemporal analysis of functional connectivity

Dynamic functional connectivity describes the phenomenon that different brain regions of similar function covary in the time and may, or may not, correspond to anatomical connections (Damoiseaux and Greicius, 2009). Regardless of the metric used to determine this association, the outcome amounts to a weighted graph, where the recorded regions are the nodes and the statistical associations constitute the edge weights (Friston, 2011), Figure 1B. For electrophysiological recordings and recently for fMRI signals (Kamiński et al., 2001; Friston et al., 2003; Mitra and Raichle, 2016), increasing effort has been directed at elucidating these weights in a directed fashion (e.g., Blinowska 2011; Bressler and Seth 2011). The Pearson correlation coefficient to assess zero-lag correlation between paired brain regions, remains the conventionally used functional connectivity metric.

**Figure 1:**
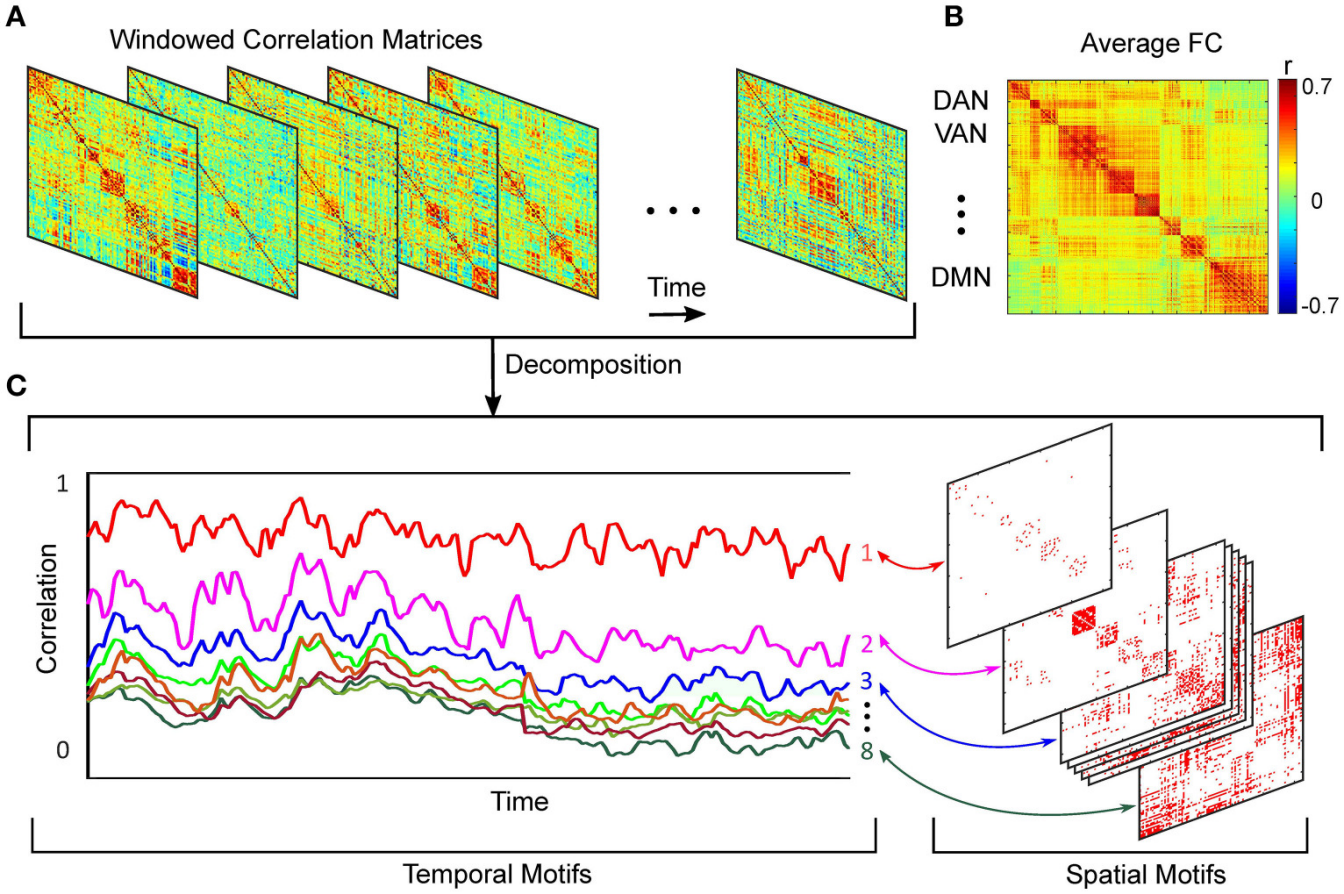
**Schematic of spatiotemporal decomposition of dynamic functional connectivity. (A)** Functional connectivity over time. **(B)** Average FC over entire scan time. **(C)** Temporal and spatial motifs obtained from spatiotemporal analyses.

Characterization of large-scale brain networks using BOLD resting-state functional connectivity MRI is typically based on the assumption of network stationarity across the duration of scan. The assumption of stationarity provides a convenient framework in which to examine and interpret results. Approaches built upon these assumptions have produced a wealth of literature expanding our knowledge of large-scale brain networks. Recent studies in humans have questioned this assumption by showing that within-network functional connectivity fluctuates on the order of seconds to minutes (Jones et al., 2012; Kiviniemi et al., 2011). Resting-state functional connectivity is not static; RSNs can exhibit non-stationary, spontaneous relationships irrespective of conscious, cognitive processing (Allen et al., 2012; Hutchison et al., 2013b). The findings imply that mechanistically important network information can be missed when using average functional connectivity as the single network measure. The changes of the functional networks of the brain are generally seen as movements from one short term state to another, rather than continuous shifts (Hutchison et al., 2013a). Many studies have shown reproducible patterns of network activity that move throughout the brain. Sliding window analysis is the most common method used in the analysis of dynamic functional connectivity. Sliding window analysis is performed by conducting analysis on a set number of scans in an fMRI session.

#### 2.4.1. Estimation of sliding window temporal covariance matrices

In order to characterize the temporal dynamics of functional connectivity, first we used a 5-frame window (11 s, adjacent non-overlapping windows) to generate covariance matrices **S**_*k*_ for each subject and at both 0 and 1.2% sevoflurane. Then, we estimated covariance from the regularized precision matrix using the graphical LASSO method by placing a penalty on the ℓ_1_ norm of the precision matrix (Θ_*k*_) to promote sparsity (Friedman et al., 2008; Monti et al., 2014). It assigns a large cost to matrices with large absolute values, thus effectively shrinking elements toward zero. For each sliding window, k, the following log-likelihood optimization problem is maximized:

(1) $$\begin{array}{l} L\left(\Theta_{k}\right)=\log\;\det\left(\Theta_{k}\right)-\text{tr}\left({\text{S}}_{k}\Theta_{k}\right)+\lambda ∥\Theta_{k}{∥}_{\ell_{1}}, \end{array}$$

where the regularization parameter, λ, allows us to balance the tradeoff between log-likelihood and the number of non-zero coefficients in the inverse covariance matrix, which was set to 0.1 in our analyses. After estimating the covariance matrix from (**1**), correlation trajectories for each region pair were calculated using a bounded Kalman filtering approach explained in Supplementary Material, Section 1. The developed filter tracked correlation dynamics, and produced estimates of correlation trajectories that are more precise than those obtained directly from measurements, i.e., obtained over sliding windows. After that, correlation time series were concatenated across subjects and both arousal states.

#### 2.4.2. Determination of spatiotemporal motifs

Using a k-means algorithm, we defined 8 orthogonal clusters with region pair membership defined by the strength of covarying correlation trajectories. We selected 8 clusters based on analysis of model fit assessed by Akaike Information Criterion (AIC) (Akaike, 1974), (Supplemental Material, Section 2). More detail on this approach is provided elsewhere (Kafashan et al., 2014). Schematic of spatiotemporal decomposition of dynamic functional connectivity is shown in Figure 1.

Our spatiotemporal motif analysis can be summarized as follow: (1) Extracting correlation trajectory for all region-pairs from each subject and at a specific arousal state, and filtering the correlation trajectories utilizing our developed bounded Kalman filter (Figure 1A). (2) Applying k-means technique to the filtered correlation trajectories to extract orthogonal motifs (Figure 1C).

Simulations on synthesized data demonstrate efficacy and utility of our approach for differentiating different patterns of dynamic fluctuations among spatially distributed clusters of brain regions with identical mean average correlation, (Supplementary Material, Figure 2).

## 3. Results

### 3.1. Widespread conservation of functional connectivity structure under sevoflurane anesthesia

**Figure 2:**
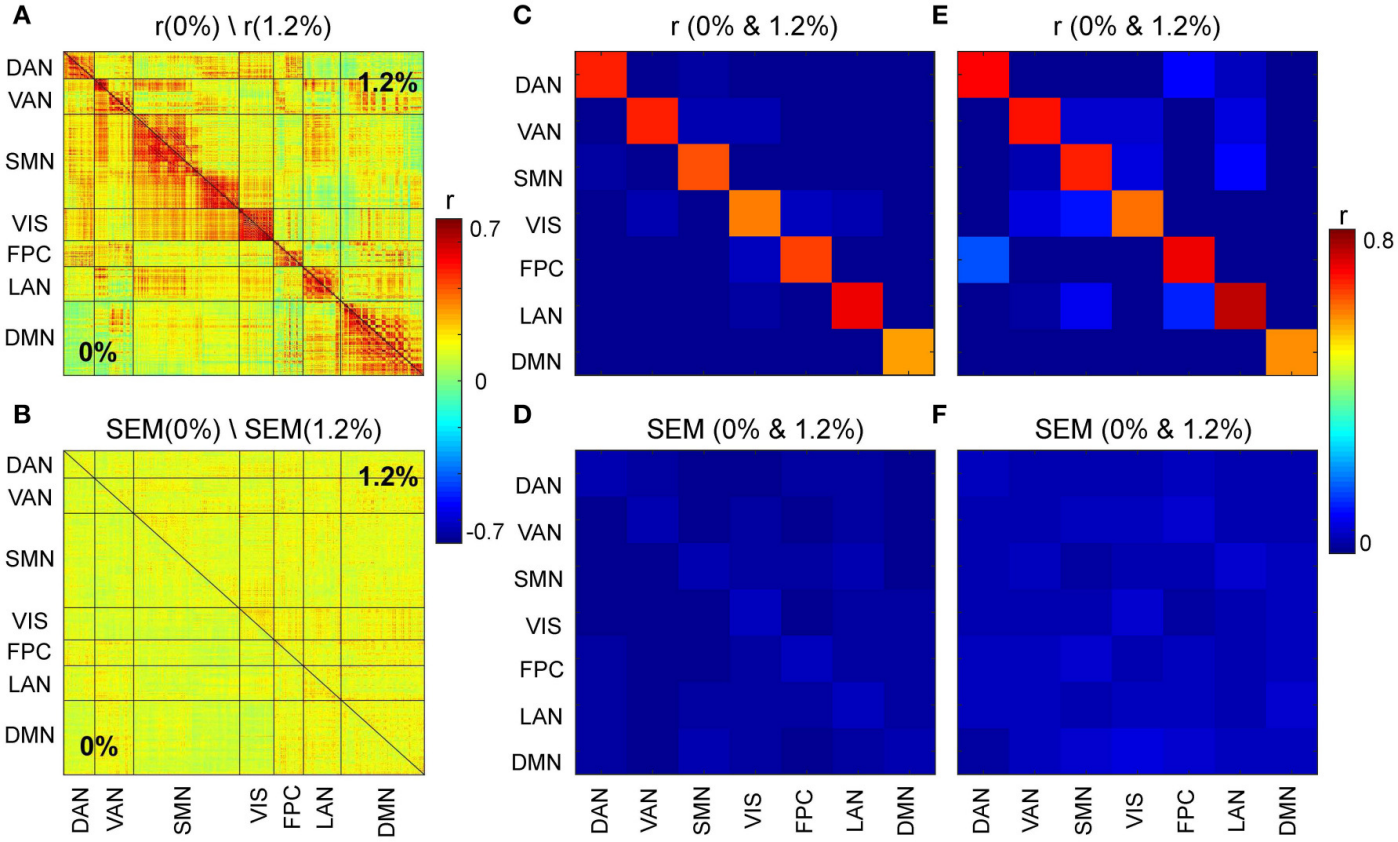
**(A)** Average correlation matrices (0 and 1.2%). **(B)** Standard error of mean (SEM) of average correlation matrices. **(C)** Similarity across average correlation matrices (0 and 1.2%) for entire data. **(D)** SEM of Similarity across average correlation matrices for entire data. **(E)** Similarity across average correlation matrices (0 and 1.2%) for subsampled data. **(F)** SEM of Similarity across average correlation matrices for subsampled data.

**Figure 3:**
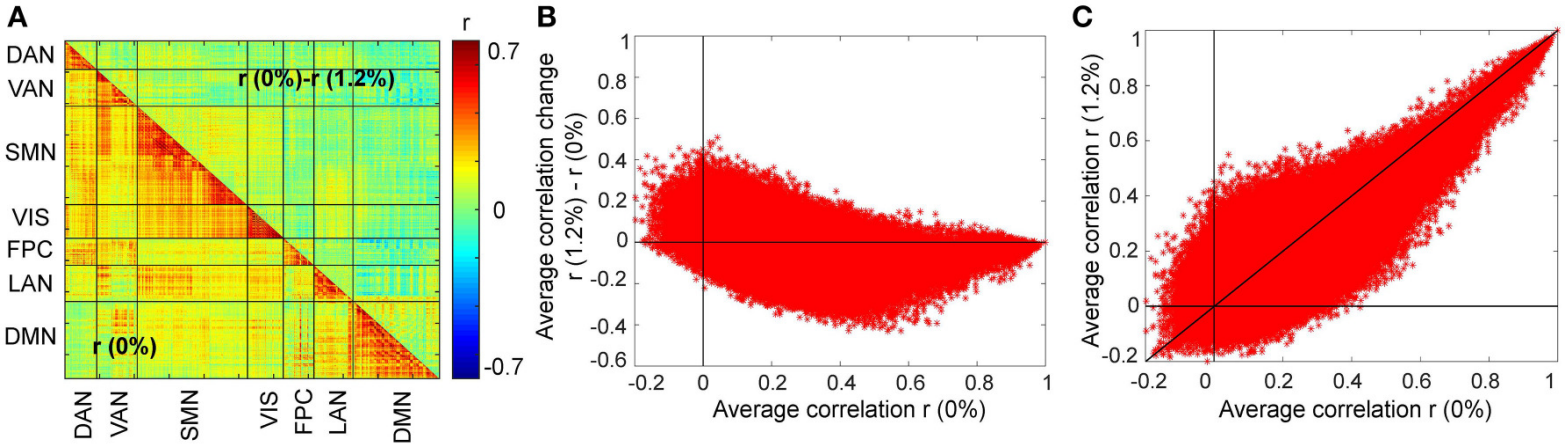
**Changes in the average FC over subjects during the transition from wakefulness (0% sevoflurane) to anesthesia (1.2% sevoflurane). (A)** The lower triangle shows the average FC over subjects at 0%, while the upper triangle demonstrates the difference of average FC at both conditions (0–1.2%) for each region pair. **(B)** Changes in the average FC for both conditions (1.2–0%) as a function of the average FC at 0%. **(C)** The average FC at 1.2% vs. average FC at 0%.

We first asked, how similar, on average, is the correlation structure during wakefulness compared to that during sevoflurane general anesthesia. This analysis complemented our earlier analysis (Palanca et al., 2015) that suggested key alterations within the DMN and VAN. Using both a simple correlation (Figure 2) for quantifying similarity across average correlation matrices (0 and 1.2%), we observed a high degree of conservation of functional connectivity within RSNs regardless of arousal state. Matrices showing the average correlation for the two conditions are similar (Figure 2A). At a region level, the standard deviation across volunteers is 0.13 and mainly 0.15. Figure 2B shows the standard error of mean (SEM) of average correlation (0 and 1.2%). We assessed the similarity between the two conditions by calculating the correlation of the mean correlation values. The overall similarity of all correlations among brains regions at 0 and 1.2% sevoflurane was 0.76. Thus, the correlated activity during wakefulness accounts for roughly 58% of that during sevoflurane general anesthesia. The greatest similarity was within RSNs, as summarized in Figure 2C, with low variance across our participants (Figure 2D). To allow feasibility for subsequent spatiotemporal analysis, we reduced dimensionality using random subsampling of all regions, 15 per RSN. Again, similarity in the average correlation structure at 0 and 1.2% sevoflurane was conserved (Figure 2E), mainly for within RSN comparisons, with variance across subjects. Figure 3A demonstrates the average FC over subjects at 0% compared to the difference of average FC at both conditions (0–1.2%). Figure 3B shows changes in the average FC for both conditions (1.2–0%) as a function of the average FC at 0%. Furthermore, average FC at 1.2% vs. average FC at 0% is depicted in Figure 3C. Note in Figure 3 that within-RSN connectivity decreases slightly during anesthesia.

### 3.2. Correlated brain activity during sevoflurane general anesthesia can be decomposed into dynamic spatiotemporal motifs

**Figure 4:**
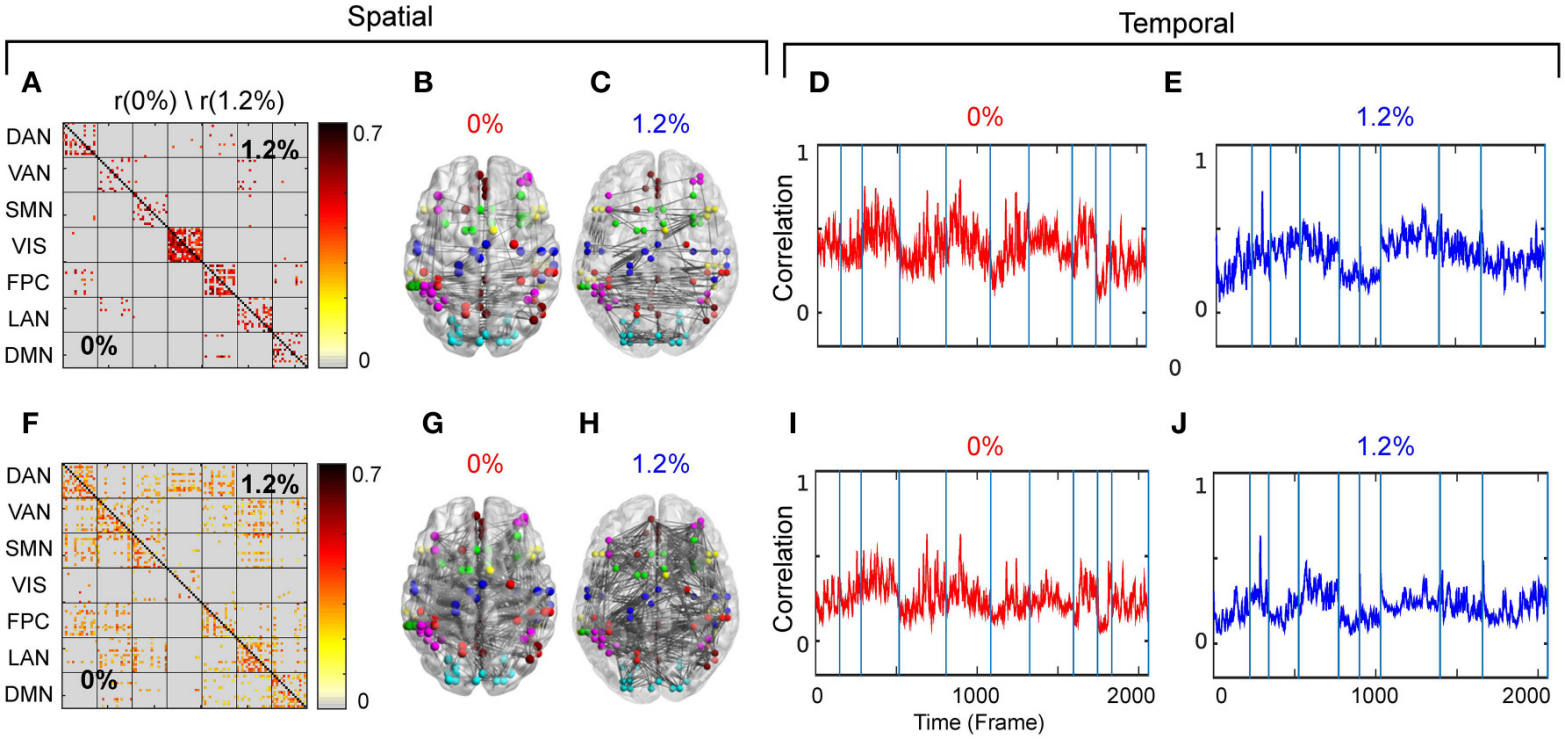
**(A)** Second spatial motif for 0 and 1.2%. **(B)** Second spatial motifs on brain surface with nodes and edges for 0%. **(C)** Second spatial motifs on brain surface with nodes and edges for 1.2%. Second temporal motif for **(D)** 0% and **(E)** 1.2%. **(F)** Third spatial motif for 0 and 1.2%. **(G)** Third spatial motifs on brain surface with nodes and edges for 0%. **(H)** Third spatial motifs on brain surface with nodes and edges for 1.2%. Second temporal motif for **(I)** 0% and **(J)** 1.2%. Blue vertical lines in the traces of the temporal motifs denote transitions of data contributed by individual participants.

Our first aim was to determine whether the temporal trajectory of correlation among pairs of brain regions respect RSN boundaries. We separately decomposed the correlations among fMRI BOLD signals acquired at 0 and 1.2% sevoflurane into eight clusters. Each cluster represents correlations between paired brain regions that follow a similar pattern over time. The resulting spatiotemporal motifs were ranked by average correlation among members. Figure 4 shows second and third spatial (Figures 4A,F) and temporal (Figures 4D,F,I,J) motifs of the decomposition.

**Figure 5:**
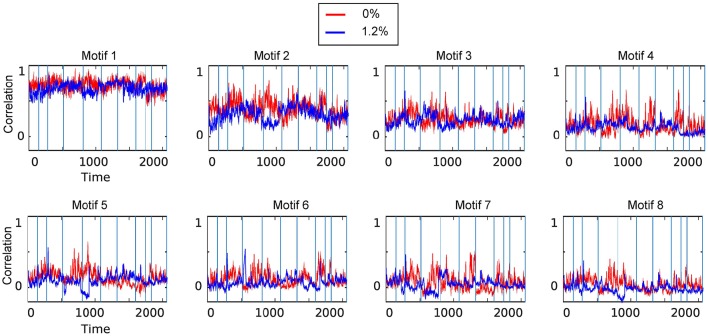
**Temporal motifs for 0 and 1.2% sevoflurane**. Blue vertical lines in the traces of the temporal motifs denote transitions of data contributed by individual participants.

**Figure 6:**
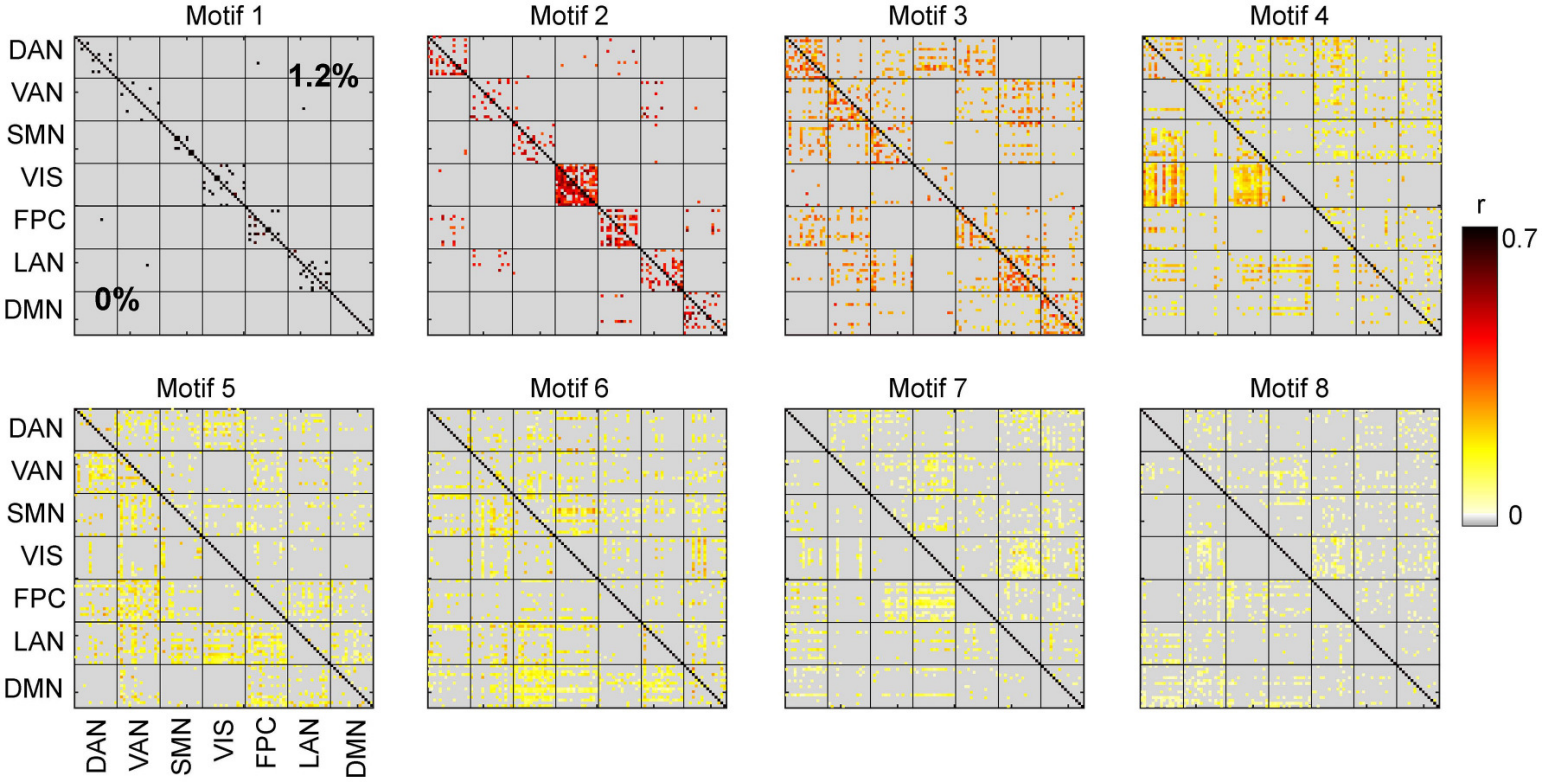
**Spatial motifs for 0 and 1.2% sevoflurane**.

We carried out spatiotemporal decomposition of the correlated brain activity measured at 0 and 1.2% sevoflurane. These motifs were determined independently as we could not assume that the motifs would be identical. Figure 5 shows all temporal motifs. All spatial motifs are shown in Figure 6. Three types of motifs were observed. The obtained spatiotemporal motifs are for one subsample. However, similar results were observed for repeated subsamples generated randomly (data not shown). Motifs 1 and 2 contained mainly within-RSN pairings with high mean correlation strength and appear relatively conserved following the transition from wakefulness to anesthetic-induced unconsciousness. Other motifs, 3–4, were composed of region pairs with intermediate correlation strength between interactions/connections within and between resting state networks. Their membership and correlation strength change between 0 and 1.2% sevoflurane. Clustering also generated motifs mainly in between-RSN region pairs of low or intermediate correlation (motifs 5–7) and a motif with negligible correlation (motif 8).

We quantified the similarity between motifs during wakefulness and 1.2% sevoflurane, compared the number of edges that were in each motif among the two conditions, and found the distributions to have no statistical difference (Chi-square test, *dF* = 49, *p* = 0.23).

The spatiotemporal motifs obtained from this approach are explained in the following sections.

#### 3.2.1. Spatiotemporal motifs that persist between wakefulness and general anesthesia

As the correlations of signals within a resting-state network show the greatest correlation (Figure 2A), one possibility is that spatiotemporal decomposition would recapitulate RSNs. Instead, our clustering generated motifs composed of brain regions spanning different RSNs but appeared to be relatively conserved between quiet wakefulness and general anesthesia, as for the motif 2 (Figure 4A). This motif is composed mainly of within-RSN pairs, suggesting that changes in correlation strength covary across RSNs. The mean correlation strength does not appear to change between wakefulness and general anesthesia. The temporal profile of average correlation over time shows marginally higher correlation and variability at 0% compared to 1.2% (0%: mean 0.39, SD 0.11; 1.2%: mean 0.34, SD 0.1, Figures 4D,E). We use the *F*-test to show that the variability in variance between two conditions is statistically significant (*F*-test, *dF*1 = 2059, *dF*2 = 1539, *p* ≪ 0.001).

In contrast, motif 3 has varying membership of intra-RSN and inter-RSN pairs (Figure 4F). As with motif 2, the temporal profile of correlations across members shows greater strength and variability at 0% sevoflurane (0%: mean 0.25, SD 8.8 × 10^−2^; 1.2%: mean 0.21, SD 7.7 × 10^−2^, *F*-test, *dF*1 = 2059, *dF*2 = 1539, *p* ≪ 0.001; Figures 4I,J). As with motifs 2 and 3 (Figures 4D,E,I,J), the correlation strength and variance in all temporal motifs is higher at 0% (Figure 5).

**Figure 7:**
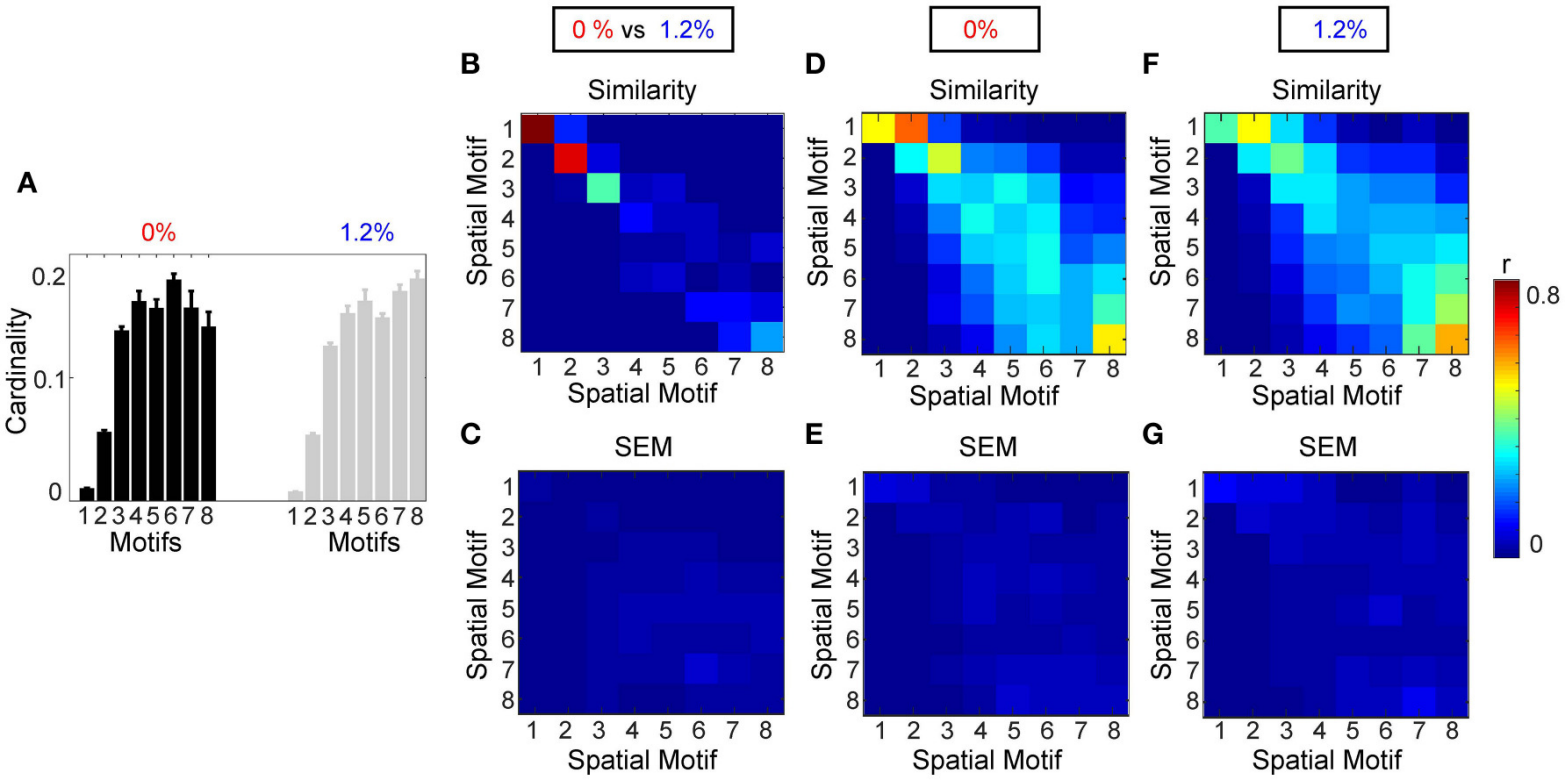
**Analysis of spatial motifs. (A)** Bar plot of the cardinality percentage of each motif for both condition (0 and 1.2%). Error bars stand for SEM. Average similarity **(B)** and SEM of average similarity **(C)** between motifs, for 8 different window size, by computing the correlation of spatial component of each motif. **(C)** Standard error of similarity between motifs. Average similarity **(D)** and SEM of average similarity **(E)** between motifs calculated from each individual at 0% sevoflurane. Average similarity **(F)** and SEM of average similarity **(G)** between motifs calculated from each individual at 1.2% sevoflurane.

Figure 7B shows the average similarity between motifs, for 8 different window sizes, by computing the correlation of the spatial component of each motif. Motifs 1, 2, 3, and 8 had the greatest similarity between wakefulness and general anesthesia. In other words, the region pairs with the strongest average correlation changed the least during the transition to anesthetic-induced unconsciousness. Standard error of the mean of similarity between motifs is shown in Figure 7C.

#### 3.2.2. Spatiotemporal motifs that vary between wakefulness and general anesthesia

Figure 7B demonstrates that there are motifs which are not similar during the transition to anesthetic-induced unconsciousness. Motifs 4–7 show very low similarity from wakefulness to anesthetic condition. An interesting result from these motifs is the observation that spatiotemporal dynamics differ between 0 and 1.2% sevoflurane mainly in interactions between resting state networks.

It can be seen in motif 4 that connections/interactions between DAN and VIS networks represent similar dynamic covariation during 0% sevoflurane, which groups them in the same motif, while such a similarity is broken in 1.2% sevoflurane. This reorganization of interactions between RSNs can be seen between VIS and SMN networks in motif 4 with intermediate strength of average correlation as well as DMN and SMN in motif 6 with intermediate average correlation.

### 3.3. Robustness as a function of parameters and intersubject variability

We also assessed the consistency of motifs as a function across individual subjects (Figures 7D–G). Figure 7A demonstrates the average cardinality of each motif over different window length. Here, eight different window lengths with 5, 10, 20, 40, 50, 60, 80, and 100 frames (with sampling time of 2.2 s per frame and adjacent non-overlapping windows) were used in the analysis. Error bars stand for SEM of the average cardinality. Figure 7D represents average similarity between motifs calculated separately from each individual during wakefulness. This figure illustrates that the motifs are robust across different subjects. In other words, high value of similarity are located mostly on diagonal elements of the similarity matrix; however, because of process noise and subject variability in some motifs, medium similarity can be seen in the off diagonal elements of the similarity matrix. Standard error of the mean of similarity in this calculation is shown in Figure 7E. In a similar way, Figure 7F demonstrates average similarity between motifs calculated separately from each individual during anesthetic-induced unconsciousness. Average similarity between motifs and the deviation from the mean for a fixed window size, 11 s, over different individuals, without concatenation, is illustrated in Supplementary Material, Figure 3.

## 4. Discussion

We quantified the extent of conservation of functional connectivity, defined by the correlation of intrinsic activity, within the cerebral cortex between wakefulness and sevoflurane general anesthesia. This was addressed in multiple ways. First, we assessed the similarity between the functional connectivity by computing correlations of the entire BOLD time-series (i.e., average correlation) for each regime. Second, we estimated how the correlation between region-pairs evolves as a function of time, thus enabling us to disambiguate region-pairs with similar average correlations into groups with different correlation dynamics. In particular, we decomposed the correlation at each moment in time into a finite set of spatiotemporal motifs whose superposition recovers the average functional connectivity. Our results indicate that the transition from 0 to 1.2% sevoflurane is associated with altered interaction between resting state networks.

### 4.1. Dynamic functional connectivity

The correlation structure of intrinsic neural activity can be decomposed into spatiotemporal motifs whose members show covariation in their temporal correlation patterns. Our clustering approach generates similar motifs for wakefulness compared to sevoflurane general anesthesia for region pairs within RSNs (motifs 1–3, 19% region pairs). Other motifs (4–6, 49% region pairs) with intermediate average correlation strength exhibit dissimilar membership between the baseline and anesthetic conditions. Finally, the large bulk of interactions between regions of different RSNs differ between motifs and have low mean correlation strength (motifs 7–8, 32% of region-pairs).

Our results complement our prior findings of susceptibility within the DMN and VAN (Palanca et al., 2015) to sevoflurane; the bulk of differences in connectivity are between RSNs and not within individual RSNs (Figure 3A).

Furthermore, our findings suggests, perhaps intuitively, that intermediate correlations between RSNs are the most sensitive to sevoflurane anesthesia. Indeed, we observe that the composition of motifs with intermediate strength of correlation is largely altered, while motifs with higher strength of average correlation are maintained at both 0 and 1.2% sevoflurane.

These observations provide a complementary characterization of temporal correlation dynamics to that posited in Hudetz et al. (2015); Barttfeld et al. (2015), wherein the correlation structure was shown to transition between a finite number of canonical patterns, referred to as microstates. These data have suggested that there may be differences in the quantity or quality of microstates following pharmacologic ablation of arousal. Here, rather than grouping the correlation patterns generated over a narrow time window, we group the temporal correlation trajectories, thus enabling a grouping of pairs whose correlation co-evolve.

### 4.2. Altered correlation dynamics in sevoflurane general anesthesia

Following an analysis of the mixed population of humans anesthetized with either propofol or sevoflurane, Huang and colleagues showed that variability in the BOLD signal was reduced in midline DMN cortical areas but greater in lateral regions (Huang et al., 2014). Furthermore, it has been shown in Huang et al. (2016) that temporal variability is coupled to signal synchronization in wakefulness while such coupling attenuates under anesthesia. Our observations suggest a mechanism for sevoflurane-induced unconsciousness involving discoordination between RSNs. Specifically, whereas within-RSN interactions appear quite robust, the motifs associated with cross-RSN interactions are fundamentally altered in their composition. Thus, those regions with co-evolving correlation in awake conditions no longer exhibit the same coordination in unconsciousness. While not a causal mechanism for unconsciousness, this observation is nonetheless consistent with the integrated information theory of (loss of) consciousness (Tononi, 2004), while also allowing for the possibility of persistent coordinated electrical activity over wide swatches of cortex during general anesthesia (Ching et al., 2010).

Our report also complements a recent study (Ranft et al., 2016) on simultaneous electroencephalography (utilizing permutation and transfer entropy) and fMRI (utilizing functional connectivity) acquired during wakefulness and multiple levels of sevoflurane general anesthesia. Our findings that strength and variability at 0% sevoflurane is higher than that at 1.2% sevoflurane for all motifs is consistent with their observations of reduced connectivity between RSNs during sevoflurane general anesthesia. At the same time, our spatiotemporal motifs may represent higher-level organization coordinating correlated activity between brain regions that vary over time. Overall, our analyses support the presence of information in transient fluctuations of the BOLD, despite its low temporal resolution compared to EEG. In this sense, our spatiotemporal motifs may represent correlated EEG signals in low-frequency bandwidths (0.01–0.1 Hz). Further investigations of simultaneous EEG and fMRI may address this possibility. Furthermore, spatiotemporal motif analysis can potentially be applied to effective connectivity in future works to characterize the interactions between different functional networks in a finer detail.

### 4.3. Concerns for non-stationarity

There are different hypotheses about non-stationarity of resting state functional connectivity over a small timescale (e.g., 1–2 min). Although there is a rich body of literature on dynamic functional connectivity which suggests meaningful correlation fluctuations in resting state networks, such fluctuations may nonetheless be a simple reflection of physiological noise, head motion, sampling variability intrinsic to small quantities of data, or bona-fide signal changes related to sleep state (Laumann et al., 2016). In our analyses, we consider a dynamic model for correlation time series, but we do not necessarily need to have non-stationary correlation trajectories to find spatial motifs with similar co-activation. Indeed, if there were no dynamicity (non-stationarity) in the correlation trajectories, each spatial motif would simply consist of region-pairs with similar (stationary) correlations. Accepting a hypothesis in which RSNs are stationary refuses the existence of discrete “microstates,” alluded to above. Our technique may substantiate either stationary or non-stationary hypotheses, and our goal is to fully describe the co-activation patterns that may reflect coordinated, time-varying brain activation.

### 4.4. Limitations

As noted in our prior report (Palanca et al., 2015), artifacts related to micromovements of the head require particular treatment in studies utilizing anesthetic agents. The regression of the whole brain global signal is critical component in our processing pipeline. The possibility remains that our findings may differ slightly, particular those involving correlation values of very low magnitude (include anticorrelations). Given that removal of this step is likely to introduce additional noise related to head motion and are not likely to affect the main findings reinforcing the flux in moderate strength correlations during the transition from wakefulness to anesthetic-induced unconsciousness. Thus, we have opted not to pursue this analysis.

We acknowledge limitations in our work that may inform future investigations. To identify spatiotemporal patterns, we used the k-means clustering technique. Though k-means clustering is an efficient and robust partitioning algorithm, it has several limitations, in particular difficulty separating clusters of different sizes and densities as well as a high susceptibility to outliers (Ertöz et al., 2003). A possible source of variation in this approach relates to the selection of the number of clusters, k. As in other investigations, we did not have an a priori value of k and instead parametrically determined the number of groups with the best fit (Supplemental Material, Figure 1). An experimental limitation of this study is the number of subjects scanned under both conditions. Though we were able to scan each subject for an extended period, increasing the number of subjects will improve the robustness of the findings.

## Ethics statement

This study was carried out under a protocol approved by the Human Research Protection Office of Washington University in St. Louis. Full written informed consent was acquired from all subjects, in accordance with the Declaration of Helsinki. A data and safety monitoring board conducted interval review of the study.

## Author contributions

Acquisition of data: BP. Analysis and interpretation of data: MK, SC, BP. Drafting of the manuscript: MK, SC, BP.

## Funding

SC holds a Career Award at the Scientific Interface from the Burroughs-Wellcome Fund. This work was partially supported by AFOSR 15RT0189, NSF ECCS 1509342 and NSF CMMI 1537015, from the US Air Force Office of Scientific Research and the US National Science Foundation, respectively. BP was supported by the Foundation for Anesthesia Education and Research (FAER MRTG-CT- 02/15/2010), the Washington University Institute of Clinical and Translational Sciences grant UL1 TR000448, subgrant KL2TR000450 from the National Center for Advancing Translational Sciences, and grant 1R21AG052821-01 from the National Institute of Aging. The content is solely the responsibility of the authors and does not necessarily represent the official views of the NIH.

### Conflict of interest statement

The authors declare that the research was conducted in the absence of any commercial or financial relationships that could be construed as a potential conflict of interest.
