# Supplementary material for: Sevoflurane Alters Spatiotemporal Functional Connectivity Motifs That Link Resting-State Networks during Wakefulness
Source: Front Neural Circuits. 2016 Dec 27;10:107. doi: 10.3389/fncir.2016.00107 (PMC5187351; doi:10.3389/fncir.2016.00107)
Supplement: Supplementary file 1 [file Presentation1.pdf]

Supplementary Material: Sevoflurane Alters Spatiotemporal Functional Connectivity Motifs That Link Resting-State Networks During Wakefulness

MohammadMehdi Kafashan,[1], ShiNung Ching,[1,2] and Ben Julian A. Palanca[3*]

[1] *Department of Electrical and Systems Engineering, Washington University in St. Louis, St. Louis, MO, USA,* [2] *Division of Biology and Biomedical Science, Washington University in St. Louis, St. Louis, MO, USA,* [3] *Department of Anesthesiology, Washington University School of Medicine in St. Louis, St. Louis, MO, USA*

Correspondence*:

Ben Julian A. Palanca

Department of Anesthesiology, Washington University School of Medicine in St. Louis, 660 S Euclid Ave, St. Louis, MO 63110, US, palancab@wustl.edu

1 DYNAMIC CORRELATION MODEL

Let $\mathbf{z}_k$, $k = 1, 2, ...$ be a vector time series of correlation values between pairs of brain regions. We assume that $\mathbf{z}_k$ arises from an underlying state $\mathbf{x}_k \in \mathbb{R}^N$ via

$$\mathbf{z}_k = \mathbf{F}^{-1}(\mathbf{x}_k), \tag{1}$$

where the function $\mathbf{F^{-1}}$ is vector-valued inverse Fisher transform which can be written as

$$\mathbf{F}^{-1}\left(\begin{bmatrix} x^1 \\ \vdots \\ x^N \end{bmatrix}\right) = \begin{bmatrix} \tanh\left(x^1\right) \\ \vdots \\ \tanh\left(x^N\right) \end{bmatrix}. \tag{2}$$

Such a function bounds the state within $[-1, 1]$ and is the standard transformation of the cross-correlation into a normally distributed variable. We assume that $\mathbf{x}_k$ obeys a standard, linear state space model of the

form

$$\mathbf{x}_k = \mathbf{A}_k \mathbf{x}_{k-1} + \mathbf{w}_{k-1},$$
$$\mathbf{y}_k = \mathbf{F}^{-1}(\mathbf{C}_k \mathbf{x}_k + \mathbf{v}_k). \tag{3}$$

Here, the state noise process $\mathbf{w}_k \in \mathbb{R}^N$ is an N-dimensional random vector with multivariate Gaussian distribution having zero mean and covariance matrix $\mathbf{Q_w} \in \mathbb{R}^{N\times N}$, the noise vector $\mathbf{v}_k \in \mathbb{R}^N$ is a zero-mean multivariate Gaussian random vector with covariance matrix $\mathbf{R_v} \in \mathbb{R}^{N\times N}$. Bounded nature of correlation coefficients result in a non-Gaussian noise for the observation vector $\mathbf{y}_k$. The covariance $\mathbf{Q_w}$ determines the extent to which correlation can change in successive measurements, while $\mathbf{R_v}$ simply characterizes measurement noise. In this sense, the vector $\mathbf{y}_k$ consists of noisy observations of pairwise correlation values. We assume that state noise and measurement noise are uncorrelated.

We seek the optimal filter for obtaining the state estimate $\hat{\mathbf{z}}_k$ in the sense of minimum mean-squared error (MMSE), i.e.,

$$\min_{\hat{\mathbf{z}}_k} \mathbb{E}\left[\|\hat{\mathbf{z}}_k - \mathbf{z}_k\|_2^2\right]. \tag{4}$$

Central to this problem is the calculation of the probability density function (p.d.f.) of the state vector at any given time k, conditioned on $\mathcal{Y}_k = \{\mathbf{y}_0, \mathbf{y}_1, \ldots, \mathbf{y}_k\}$, the set of all the past observations. The presence of the nonlinearity (2) complicates this calculation, but only slightly since $\mathbf{F}$ is smooth and invertible. Thus, it follows immediately that we can obtain a surrogate measurement

$$\mathbf{d}_k = \mathbf{F}(\mathbf{y}_k), \tag{5}$$

such that $\mathbf{d}_k$ is linear in the state $\mathbf{x}_k$. Note that (1) directly yields the p.d.f. of $\mathbf{z}_k$ from that of $\mathbf{x}_k$. Thus, the problem (4) reduces to the classical Kalman filter to obtain the (Gaussian) p.d.f. of $\mathbf{x}_k$ given $\mathcal{Y}_k$ (Kalman, 1960b,a), based on the measurements $\mathbf{d}_k$.

It follows directly from (3)-(5) that the p.d.f. $p(\mathbf{y}_k|\,\mathbf{x}_k)$ can be written as

$$p\left(\mathbf{y}_k|\,\mathbf{x}_k\right) = \frac{1}{|\mathbf{J}_k|} p\left(\mathbf{v}_k = \mathbf{F}(\mathbf{y}_k) - \mathbf{C}_k \mathbf{x}_k\right), \tag{6}$$

where $p\left(\mathbf{v}_k = \mathbf{F}(\mathbf{y}_k) - \mathbf{C}_k \mathbf{x}_k\right)$ is p.d.f. of $\mathbf{v}_k$, a zero-mean multivariate Gaussian with covariance matrix $\mathbf{R_v}$. A standard Bayesian approach thus yields the posterior density $p\left(\mathbf{x}_k|\,\mathcal{Y}_k\right)$, which is Gaussian with

covariance matrix ($\boldsymbol{\Sigma}$) and mean (μ) as

$$\begin{aligned}\boldsymbol{\Sigma} &= \left(\mathbf{P}_{k|k-1}^{-1} + \mathbf{C}_k{}^T \mathbf{R}_{\mathbf{v}}^{-1} \mathbf{C}_k\right)^{-1}, \\ \mu &= \boldsymbol{\Sigma} \times \left(\mathbf{P}_{k|k-1}^{-1} \hat{\mathbf{x}}_{k|k-1} + \mathbf{C}_k{}^T \mathbf{R}_{\mathbf{v}}^{-1} \mathbf{F}(\mathbf{y}_k)\right).\end{aligned} \tag{7}$$

The Kalman update equations are thus

$$\begin{aligned}\hat{\mathbf{x}}_{k|k-1} &= \mathbf{A}_k \hat{\mathbf{x}}_{k-1|k-1}, \\ \mathbf{P}_{k|k-1} &= \mathbf{A}_k \mathbf{P}_{k-1|k-1} \mathbf{A}_k^T + \mathbf{Q}_{\mathbf{w}}, \\ \hat{\mathbf{x}}_{k|k} &= \left(\mathbf{P}_{k|k-1}{}^{-1} + \mathbf{C}_k{}^T \mathbf{R}_{\mathbf{v}}{}^{-1} \mathbf{C}_k\right)^{-1} \\ &\quad \times \left(\mathbf{P}_{k|k-1}{}^{-1} \hat{\mathbf{x}}_{k|k-1} + \mathbf{C}_k{}^T \mathbf{R}_{\mathbf{v}}{}^{-1} \mathbf{F}(\mathbf{y}_k)\right), \\ \mathbf{P}_{k|k} &= \left(\mathbf{P}_{k|k-1}{}^{-1} + \mathbf{C}_k{}^T \mathbf{R}_{\mathbf{v}}{}^{-1} \mathbf{C}_k\right)^{-1}.\end{aligned} \tag{8}$$

In the subsequent results, we assume an initial multivariate Gaussian prior $\mathbf{x}_{0|-1}$ with mean $\hat{\mathbf{x}}_{0|-1}$ and covariance of $\mathbf{P}_{0|-1}$. We note that, for the problem (4), (8) returns the optimal estimate. After obtaining state estimate $\hat{\mathbf{x}}_{k|k}$, the correlation coefficients $\hat{\mathbf{z}}_k$ are approximated using fourth order Taylor expansion of (1) around $\hat{\mathbf{x}}_{k|k}$ as

$$\begin{aligned}\hat{\mathbf{z}}_k &= \tanh(\hat{\mathbf{x}}_{k|k}) - \tanh(\hat{\mathbf{x}}_{k|k}) * \text{sech}^2(\hat{\mathbf{x}}_{k|k}) * \text{diag}(\mathbf{P}_{k|k}) + \\ &\frac{1}{24}(22\tanh(\hat{\mathbf{x}}_{k|k}) * \text{sech}^4(\hat{\mathbf{x}}_{k|k}) - 2\sinh(3\hat{\mathbf{x}}_{k|k}) * \text{sech}^5(\hat{\mathbf{x}}_{k|k})) * 3\text{diag}(\mathbf{P})_{k|k}^2,\end{aligned} \tag{9}$$

where all the multiplication and powers of vectors are element-wise operations. Also, diag() operator applies to a square matrix and returns a vector containing the diagonal elements of the matrix.

A final obstacle in using the filter equations (8) is knowing the noise parameters that specify the state-space model. Since such information is difficult to define a priori, a principled way to update them alongside the state estimates is required. Here, we face the challenge of estimating noise parameters alongside our online filtering. To address this issue we use an EM algorithm (Ghahramani and Hinton, 1996; Shumway and Stoffer, 2010) to jointly estimate the state and model parameters from observed correlation data. Assuming diagonal covariance matrices for model parameters, we can decouple the update procedures for each edge, resulting in a reduction of the computational burden typically associated with EM methods. Briefly speaking, after initializing the model parameter (process and measurement noise), we perform the E-step, filtering and smoothing, and then updating the parameter in the M-step. The E and M steps alternate

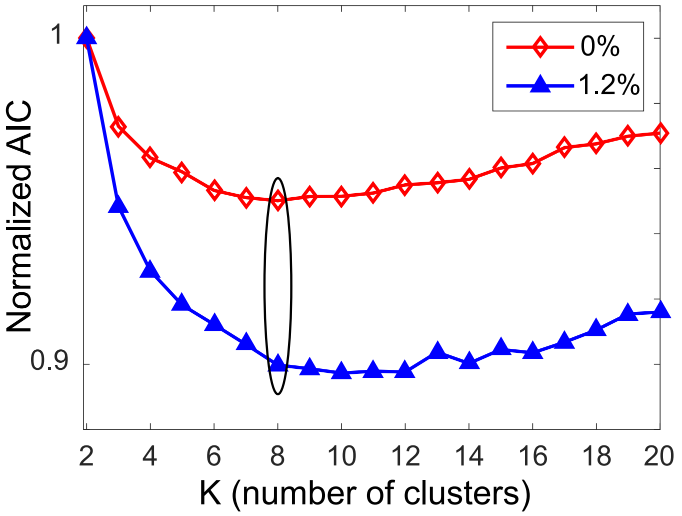

Figure 1: **Normalized AIC versus number of cluster K.** AIC values are normalized to have maximum value of unity for each condition.

to convergence. In order to obviate computational issues associated with this algorithm, a maximum iteration number is set alongside the convergence criterion.

2 DETERMINING THE NUMBER OF MOTIFS USING AIC

Selecting the number of clusters, K, in unsupervised learning is one of the key problem. The correct choice of K is often ambiguous. There exists several heuristic methods to facilitate the selection of k such as "The Elbow Method" by (Thorndike, 1953) and a technique based on the use of hierarchical clustering by (Salimi-Khorshidi et al., 2011). Another set of approaches for determining k are information criteria methods. In this paper, we adopt the Akaike information criterion (AIC) (Akaike, 1974) approach. AIC rewards goodness of fit, assessed by the likelihood function, but it also includes a penalty that is an increasing function of K. Figure 1 shows AIC versus K for both 0% and 1.2% vol sevoflurane. In this plot, AIC values are normalized to have maximum value of unity for each condition. It can be seen from this figure that K = 8 is the optimal choice of K for both condition.

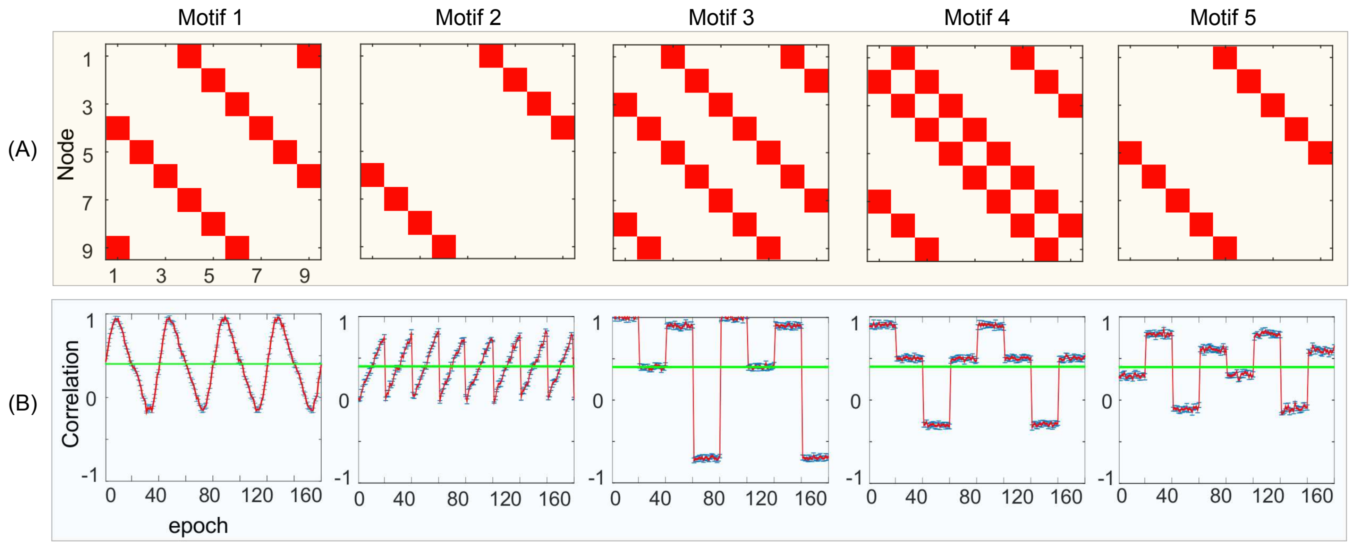

Figure 2: **(A)** Spatial motifs and **(B)** temporal correlation trajectories associated with each cluster for synthesized data. Green line shows the average correlation over time and space for each cluster.

3 SPATIOTEMPORAL ANALYSES OF SYNTHESIZED DATA

Here, we demonstrate the efficacy and utility of the approach in a simple example of a data set with 9 locations and 160 time windows. We assume there exists 5 spatial motifs shown in Figure 2A and each motif has the same temporal correlation trajectory with the same mean over time (Green lines shown in Figure 2B). Before decomposing the correlation trajectories, we added noise drawn from uniform distribution inside interval $(-0.1, 0.1)$ to all region pair correlations over time. Figure 2 shows spatiotemporal decomposition of correlation time series. Spatial and temporal motifs are shown in Figures 2A and 2B, respectively. It can be conclude from these plots that temporal information can play an important role for extracting meaningful motifs even though if all the region pairs have the same static correlation value.

4 ROBUSTNESS AS A FUNCTION OF INTERSUBJECT VARIABILITY

To see how consistent the spatiotemporal motifs are for different conditions (0% and 1.2%), we look at how spatial motifs at 0% and 1.2% obtained from each individual, without concatenating data from all subjects for each condition, are similar. Figure 3A demonstrates the average cardinality of each motifs over different individual. Error bars stand for SEM of the average cardinality. Figure 3B represents average similarity

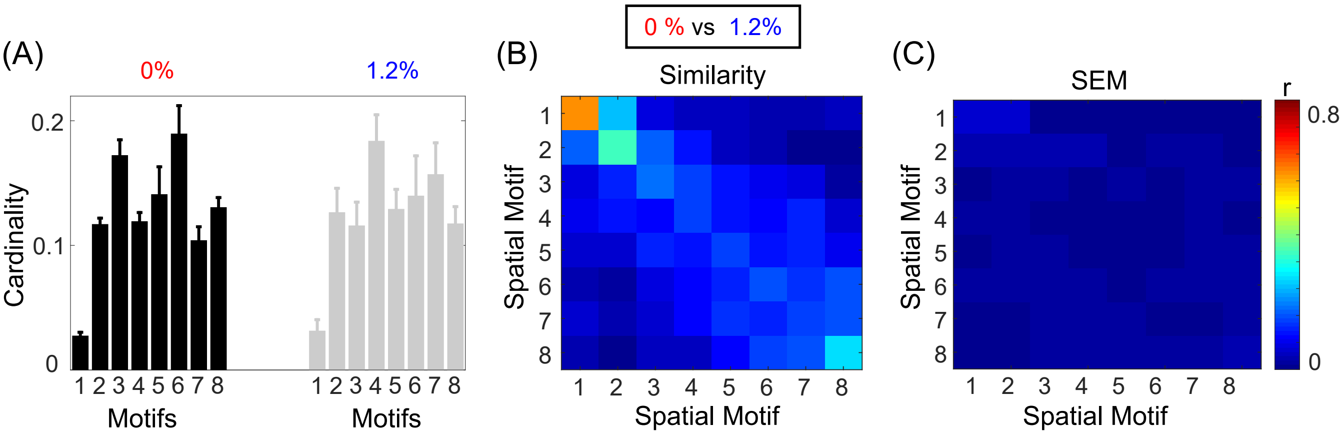

Figure 3: **(A)** Bar plot of the cardinality percentage of each motif for both condition (0% and 1.2%) for a fixed window size, 11 seconds, over different individuals. Error bars stand for SEM. Average similarity **(B)** and SEM of average similarity **(C)** between motifs over different individuals, by computing the correlation of spatial component of each motif.

between motifs at 0% and 1.2% obtained from each individual for 11 seconds window size. Standard error of the mean of the average similarity is shown in Figure 3C. We can see from this figures that the similarity between first, second and eighth motifs are robust over different individual.

REFERENCES

Akaike, H. (1974). A new look at the statistical model identification. *IEEE Transactions on Automatic Control*, 19(6):716–723.

Ghahramani, Z. and Hinton, G. E. (1996). Parameter estimation for linear dynamical systems. Technical report, Technical Report CRG-TR-96-2, University of Totronto, Dept. of Computer Science.

Kalman, R. E. (1960a). Contributions to the theory of optimal control. *Bol. Soc. Mat. Mexicana*, 5(2):102–119.

Kalman, R. E. (1960b). A new approach to linear filtering and prediction problems. *Journal of basic Engineering*, 82(1):35–45.

Salimi-Khorshidi, G., Nichols, T. E., Smith, S. M., and Woolrich, M. W. (2011). Using gaussian-process regression for meta-analytic neuroimaging inference based on sparse observations. *Medical Imaging, IEEE Transactions on*, 30(7):1401–1416.

Shumway, R. H. and Stoffer, D. S. (2010). *Time series analysis and its applications: with R examples*. Springer Science & Business Media.

Thorndike, R. L. (1953). Who belongs in the family? *Psychometrika*, 18(4):267–276.
